# Water Purification Using Choline-Amino Acid Ionic Liquids: Removal of Amoxicillin

**DOI:** 10.1021/acs.iecr.4c01002

**Published:** 2024-05-21

**Authors:** Pedro Velho, Catarina Lopes, Eugénia A. Macedo

**Affiliations:** † LSRE-LCM—Laboratory of Separation and Reaction Engineering—Laboratory of Catalysis and Materials, Faculty of Engineering, University of Porto, Rua Dr. Roberto Frias, 4200-465 Porto, Portugal; ‡ ALiCE—Associate Laboratory in Chemical Engineering, Faculty of Engineering, University of Porto, Rua Dr. Roberto Frias, 4200-465 Porto, Portugal

## Abstract

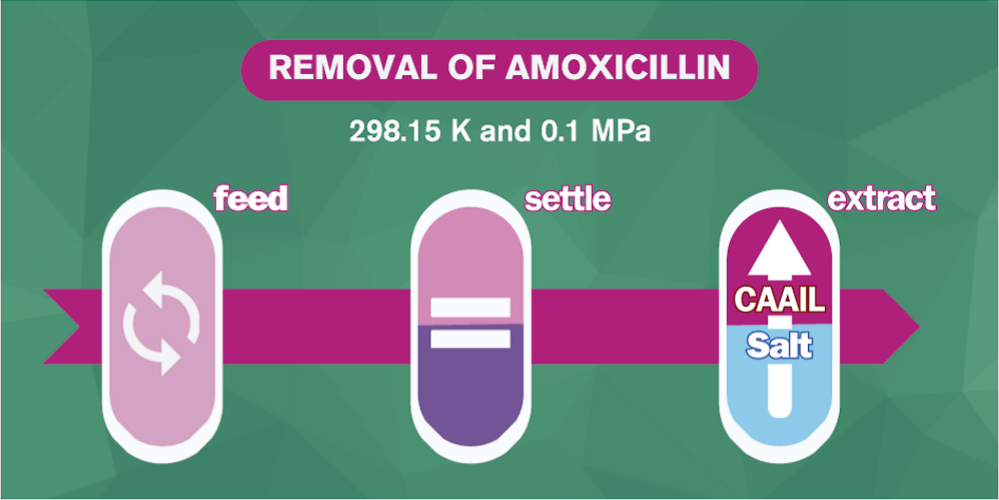

Antibiotics are the main active pharmaceutical ingredients (APIs) for the treatment and prevention (prophylaxis) of bacterial infections, for which they are essential for health preservation. However, depending on the target bacterial strain, an efficient treatment may imply weeks of continuous intake of antibiotics, whose unmetabolized fraction ends up in the wastewater system by human and animal excreta. The presence of these chemical compounds in wastewater is known to damage aquatic ecosystems and cause antibiotic resistance of pathogenic agents, which threatens the future application of these medicines. Aqueous two-phase systems (ATPSs), an emergent extraction technology for biomolecules such as proteins and vitamins, could provide more eco-friendly and cost-effective extractive alternatives given their nontoxicity and low energetic requirements. Moreover, choline-amino acid ([Ch][AA]) ionic liquids (also known as CAAILs or ChAAILs) are considered one of the greenest classes of ionic liquids due to their favorable biocompatibility, biodegradability, and ease of chemical synthesis. In this work, partition studies of amoxicillin were performed in three ATPSs containing dipotassium hydrogen phosphate (K_2_HPO_4_) and the CAAILs (cholinium l-alaninate, [Ch][Ala]; cholinium glycinate, [Ch][Gly]; and cholinium serinate, [Ch][Ser]) at 298.15 K and 0.1 MPa. To better characterize the extract and reduce errors in quantification, the effect of pH on the intensity and stability of the UV–vis spectra of amoxicillin was studied prior to the partition studies, and computational chemistry was used to validate the molecular structure of the synthesized ionic liquids. During experimental determinations, it was observed that the extraction of amoxicillin was favored by less polar ionic liquids, achieving maximum partition coefficients (*K*) and extraction efficiencies (*E*) of *K* = (16 ± 6)·10^1^ and *E* / % = 97 ± 2, respectively, for {[Ch][Gly] (1) + K_2_HPO_4_ (2) + Water (3)} in the longest tie-line.

## 1 Introduction

Antibiotics are an important class of pharmaceuticals used to prevent illnesses and infections in humans and other animals.^1^ However, nonmetabolized antibiotics are expelled through urine and feces and end up in manure and water bodies.^2^ As a result, antibiotics have been found in aquatic settings such as surface water and groundwater, appearing in sewage treatment facilities and in drinking water.^3^

One of the most prescribed antibiotics in the world is amoxicillin,^4^ which belongs to the β-lactam family.^5^ This active pharmaceutical ingredient (API) is frequently used to treat bacterial skin infections, pneumonia, and urinary tract infections.^6^ Given the widely reported presence of this antibiotic in wastewater,^7^ it is included in the European Surface Water Watch List, under the European Union Water Framework Directive (Decision 2022/1307). While human and animal excreta are the main sources of amoxicillin in aquatic environments, other significant sources include hospital and home sewage, pharmaceutical industry effluents, and leachates from landfills.^6^ The presence of amoxicillin in the aquatic environment has been reported to result in extreme allergies in fresh algae, plankton, micro- and macrophytes, and fishes,^8^ so it is necessary to develop efficient extractive methodologies for this pollutant.

Ozonation,^9^ advanced oxidation processes, such as the Fenton^10^ or photo-Fenton system,^11^ photocatalysis using TiO_2_,^12^ membrane processes^13^ (namely, ultrafiltration, nanofiltration, and osmosis) are some examples of novel treatment technologies to treat secondary effluents. Moreover, aqueous two-phase systems (ATPSs), also known as aqueous biphasic systems, constitute another promising extraction technology given their good potential for continuous operation, simple scaling-up, eco-friendliness, and mildness of extractive conditions.^14,15^

Ionic liquids (ILs), also known as fused salts or molten salts, are a class of ionic compounds with a melting point below 100 °C at ambient pressure,^16^ which are often applied in ATPSs. Generally, ILs present high viscosity, low vapor pressure, tunable solubility, high thermal stability, and extremely low corrosivity in comparison to mineral acids and bases.^17^ Despite ILs being considered “green solvents”, the most common cations (*e.g.*, imidazolium and pyridinium) and anions (often fluorine-based) show scarce biodegradability and biocompatibility.^18,19^ Therefore, ILs based on amino acids and choline, which are nontoxic and biodegradable, were developed, which are known as choline-amino acid ([Ch][AA]) ILs [or choline-amino acid ILs (CAAILs)].^20^ CAAILs are seen as the most promising and secure ILs since they present lower toxicity, higher biocompatibility, and higher biodegradability, as well as more abundant raw materials.^21^

In literature, the extraction of amoxicillin in ATPSs was mainly carried out using polymers such as poly(ethylene glycol) (PEG) with 1500^22^ and 6000^23^ g·mol^–1^, inorganic salts such as K_2_HPO_4_^23^ and Li_2_SO_4_,^22^ and organic salts such as Na_2_C_4_H_4_O_6_ (disodium tartrate)^22^ and Na_3_C_6_H_5_O_7_ (trisodium citrate).^22^ Nevertheless, no studies are known involving the application of CAAILs in the removal of this API. Therefore, the main goal of this work was to conduct the extraction of amoxicillin employing CAAIL-based ATPSs at 298.15 K and 0.1 MPa, in the search for more sustainable liquid–liquid extraction approaches for this pharmaceutical pollutant.

## 2 Experimental Procedure

### 2.1 Materials

Table 1 presents the chemicals used in this work and their respective commercial supplier, purities, Chemical Abstracts Service (CAS) number, and chemical formula. All chemicals were used without further purification steps.

**Table 1:** Chemicals Used with Respective Commercial Suppliers, Purities, CAS Numbers, and Chemical Formulas

chemical	supplier	purity / %^a^	CAS	chemical formula
acetic acid	Merck	> 99.8	64-19-7	CH_3_COOH
amoxicillin trihydrate	Tokyo Chemical Industry	> 98	61336-70-7	C_16_H_19_N_3_O_5_S·3H_2_O
choline chloride	VWR Chemicals	> 99	67-48-1	C_5_H_14_NOCl
dipotassium hydrogen phosphate	Merck	> 99	7758-11-4	K_2_HPO_4_
ethanol	Sigma-Aldrich	> 99	64-17-5	CH_3_CH_2_OH
glycine	Merck	> 99.7	56-40-6	C_2_H_5_NO_2_
l-alanine	Fluka	> 99	302-72-7	C_3_H_7_NO_2_
l-serine	Sigma	> 99	56-45-1	C_3_H_7_NO_3_
potassium hydroxide	Merck	> 99	1310-58-3	KOH
sodium hydroxide	Merck	> 99	1310-73-2	NaOH
purified water	VWR Chemicals	> 99	7732-18-5	H_2_O

a Provided by the supplier in mass percentage.

### 2.2 Apparatus and Procedure

#### 2.2.1 Apparatus

In this work, mass (*m*) was assessed with an ADAM AAA 250L balance with standard measurement uncertainty of 10^–4^ g, pH was measured with a VWR pH 1100 L pH meter with standard measurement uncertainties of 0.001 and 0.1 K, liquid density (ρ) was determined with an Anton Paar DSA-4500 M densimeter with standard measurement uncertainties of 5 × 10^–5^ g·cm^–3^ and 0.01 K, ultraviolet–visible (UV–vis) absorbance (*A*) was determined with a Thermo Scientific Varioskan Flash spectrophotometer with a standard measurement uncertainty of 10^–4^, and the Fourier-transform infrared (FTIR) spectra were assessed with a PerkinElmer Spectrum Two FT-IR spectrometer, with a relative measurement uncertainty of 0.0001. The Anton Paar DSA-4500 M densimeter was calibrated using pure water, and according to the technical manual, while the VWR pH 1100 L pH meter was rectified with standard solutions of pH = 4.00, 7.00, and 11.00. Moreover, in the partition studies, equilibrium temperature (*T*) was kept at 298.15 K using a thermoregulated water bath OvanTherm MultiMix BHM5E with a measurement uncertainty of 0.1 K. This temperature was rechecked with a glass thermometer with a standard measurement uncertainty of 0.01 K.

#### 2.2.2 Synthesis of the [Ch][AA] ILs

**Figure 1:**
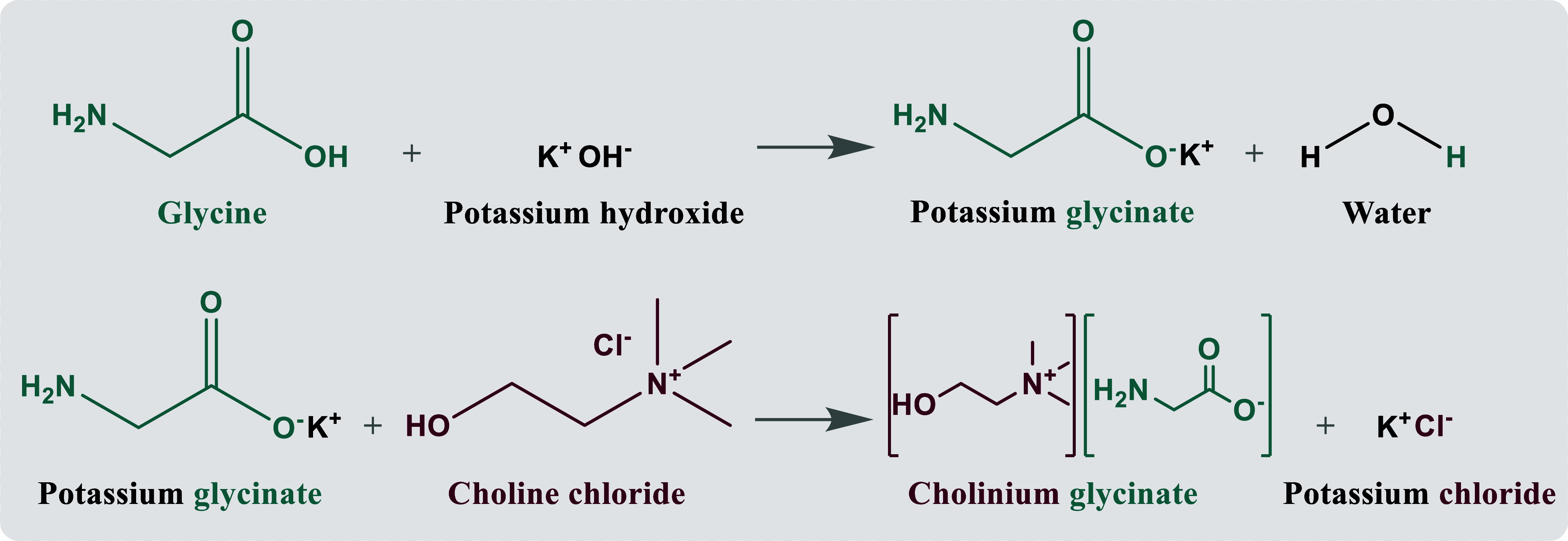
Synthesis of CAAIL cholinium glycinate, [Ch][Gly].

In the synthesis of the CAAILs, which was based on a previous work,^20^ each amino acid (l-alanine, glycine, or l-serine) and an excess of potassium hydroxide (KOH) were mixed with 500 g of ethanol in a stirred reactor for 3 h at 303.15 K and 0.1 MPa until complete dissolution of KOH. Then, choline chloride was added in stoichiometric proportions regarding the amino acid salt, and the mixture was stirred continuously for approximately 12 h (overnight) at the same temperature and pressure. Afterward, the formation of a white suspension was noticed due to the lower solubility of potassium chloride (KCl) in ethanol (0.034% in mass at 298.15 K^24^) compared to the one in water (26.476% in mass at 298.15 K^24^). To separate the synthesized CAAIL from residual KCl, the solution was filtered under vacuum. Moreover, to remove the solvents (water and ethanol), the resulting solution was distilled by using an IKA RV 10 Rotary Evaporator (323.15 K for 3 days). As example, Figure 1 shows the reaction route for the synthesis of cholinium glycinate ([Ch][Gly]). The syntheses of the other CAAILs (cholinium alaninate, [Ch][Ala], and cholinium serinate, [Ch][Ser]) were conducted in analogous manners.

#### 2.2.3 Structural and Physical Characterization of [Ch][AA] ILs

The chemical structures of the obtained CAAILs were studied by means of FTIR spectroscopy within the wavenumber of 4000–400 cm^–1^, using a PerkinElmer spectrum two FT-IR spectrometer. Then, the determined structures were confirmed with computational chemistry using density functional theory, following previous works of the research group.^20,25^ More precisely, the IR spectrum of each IL was predicted using the GAUSSIAN G09.63 software^26^ with the combination B3LYP hybrid functional^27^/6-311+G(*,*) basis set.^28^

On the other hand, to further validate the chemical synthesis, the liquid densities (ρ) of the synthesized CAAILs were measured, from 288.15 to 323.15 K, with an Anton Paar DSA-4500 M densimeter and compared with data from literature. Prior to the FTIR and liquid density measurements, the water content of the ILs was assessed by freeze-drying, and it was found to be less than 1% (in mass). This water contamination was accounted for in the calculation of final mass fractions.

#### 2.2.4 Influence of pH on UV–vis Absorbance

Solid amoxicillin was dissolved in water, and several stock solutions were prepared with a concentration of about 3.20 × 10^–4^ g g^–1^ and with different pH values (3.12, 4.85, 8.07, and 12.22). The pH values of these solutions were adjusted by adding droplets of 0.5 M aqueous solutions of sodium hydroxide (NaOH) or acetic acid (CH_3_COOH), and concentrations were recalculated, keeping in mind the added amount of pH adjusters. Then, an UV–vis absorbance scanning was performed for each solution, from 200 to 600 nm, at 298.15 K, using the Thermo Scientific Varioskan Flash UV–Vis spectrophotometer. Moreover, to evaluate their stability with time, the UV–vis spectra of all solutions were re-evaluated after 3 days. Then, after having found a stable local maximum in UV–vis absorbance (272 nm), a calibration curve was determined for amoxicillin at the characteristic pH of the studied ATPSs (pH ≅ 12) by evaluating the absorbance of samples with known concentration. Then, the absorbance of blanks (plate and pure water) was subtracted to the obtained values, and a first-degree fitting was conducted.

#### 2.2.5 Liquid–Liquid Extraction of Amoxicillin

In this work, 3 different ATPSs were studied at 298.15 K and 0.1 MPa: {cholinium alaninate or cholinium glycinate or cholinium serinate (1) + dipotassium hydrogen phosphate (2) + water (3)}. The liquid–liquid equilibria of these ATPSs were determined in the previous work,^29^ as Table 2 shows.

**Table 2:** Phase Composition (in Mas Percentage), and Corresponding Tie-Line Length, for the ATPSs {[Ch][Ala] or [Ch][Gly] or [Ch][Ser] (1) + K_2_HPO_4_ (2) + Water (3)} at 298.15 K and 0.1 MPa.^29^^a^

	feed			separation			
tie-line	[CAAIL]_feed_ / %	[salt]_feed_ / %	TLL / %	phase	[CAAIL] / %	[salt] / %	pH
{[Ch][Ala] (1) + K_2_HPO_4_ (2) + Water (3)}							
1	29.860	34.197	82.179	top	61.969	3.862	13.64
				bottom	1.683	61.596	13.30
2	31.927	30.668	78.036	top	59.007	4.300	13.63
				bottom	2.693	59.670	13.18
3	36.098	23.006	69.754	top	54.085	5.917	13.57
				bottom	4.038	55.757	13.17
4	40.002	14.046	57.642	top	46.535	8.790	13.55
				bottom	6.681	50.929	13.10
{[Ch][Gly] (1) + K_2_HPO_4_ (2) + Water (3)}							
1	31.369	39.990	95.127	top	66.992	3.717	12.72
				bottom	1.683	72.883	12.84
2	33.104	32.910	85.053	top	61.127	5.076	12.60
				bottom	2.693	66.877	12.78
3	36.111	24.979	73.637	top	53.912	7.374	12.36
				bottom	4.038	61.550	11.94
4	39.284	16.661	58.908	top	46.376	10.748	12.24
				bottom	6.681	54.274	11.71
{[Ch][Ser] (1) + K_2_HPO_4_ (2) + Water (3)}							
1	35.085	40.403	102.347	top	76.288	3.453	12.88
				bottom	1.911	73.758	13.38
2	39.076	28.915	82.944	top	63.161	7.416	12.38
				bottom	3.447	64.983	12.33
3	40.756	22.998	69.028	top	54.695	11.233	12.33
				bottom	5.468	59.622	12.34
4	43.071	19.933	65.004	top	52.080	12.512	12.32
				bottom	5.894	58.255	12.09

a The standard measurement uncertainties (*u*) are *u*(*T*) = 0.1 K, *u*(*P*) = 2 kPa, *u*(pH) = 10^–2^ and *u*([CAAIL]) = *u*([salt]) = 10^–3^.

Triplicate 20 g samples of these feed compositions were prepared in vials by pipetting pure water and the respective CAAIL and an aqueous solution of dipotassium hydrogen phosphate (K_2_HPO_4_, 60.18% in mass). During preparation, 1 mL of the reported water content was replaced by 1 mL of amoxicillin solution (1.90 × 10^–3^ g g^–1^). Then, these samples were stirred in a VWR VV3 vortex for 1 min and in an IKA RO 10 P magnetic stirrer for 8 h and left to settle overnight (approximately 12 h) until equilibrium was attained. Afterward, the top and bottom phases were separated with syringes and weighed in an ADAM AAA 250L balance. Moreover, the UV–vis absorbances, the pH values, and the liquid densities of the two phases of each tie-line were assessed, by this order.

To assess phase separation, phase mass loss (*L*_m_) was calculated for each tie-line using
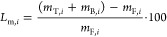
1where *i* is the number of tie-line, *m*_F_ is the mass of feed mixture, *m*_T_ is the mass of the top phase, and *m*_B_ is the mass of the bottom phase.

Then, using the determined UV–vis absorbance calibration curve, the concentration of amoxicillin in each phase was obtained, and the respective partition coefficient (*K*) was calculated for each tie-line using eq 2. It must be noted that, before calculating concentrations, the absorbance of the blanks (tie-line composition without replacing 1 mL of water by 1 mL of amoxicillin solution) was subtracted from the measured absorbances.
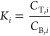
2where *i* refers to the number of tie-line and *C*_T_ and *C*_B_ to the calculated amoxicillin concentrations in the top and bottom phases, respectively.

Following other works of the research group,^20,30^ the partition coefficients were validated by performing a mass balance to the added solute. Hence, amoxicillin mass losses (*L*_s_) in quantification were determined in each tie-line using eq 3.
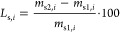
3where *i* is the number of tie-line, *m*_s1_ is the added mass of amoxicillin in the system for each feed, and *m*_s2_ is the quantified mass of amoxicillin, which was calculated using eq 4.
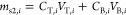
4where *i* is the number of tie-line, *C* is the concentration of amoxicillin obtained from the UV–vis absorbance calibration curve (in g·mL^–1^), T and B refer to the top and bottom phases, respectively, and *V* is the phase volume, which was calculated using eq 5.
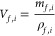
5where *i* is the number of tie-line, *f* refers to the top or bottom phase, *m* is the measured phase mass, and *ρ* is the measured liquid density.

Besides the partition coefficients, another performance indicator of extraction was determined: the extraction efficiency (*E*). *E* refers to the percentage of target solute which was recovered by the top phase in each system for each feed composition and was calculated by using eq 6.
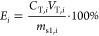
6

## 3 Results and Discussion

### 3.1 Characterization of [Ch][AA] ILs

**Figure 2:**
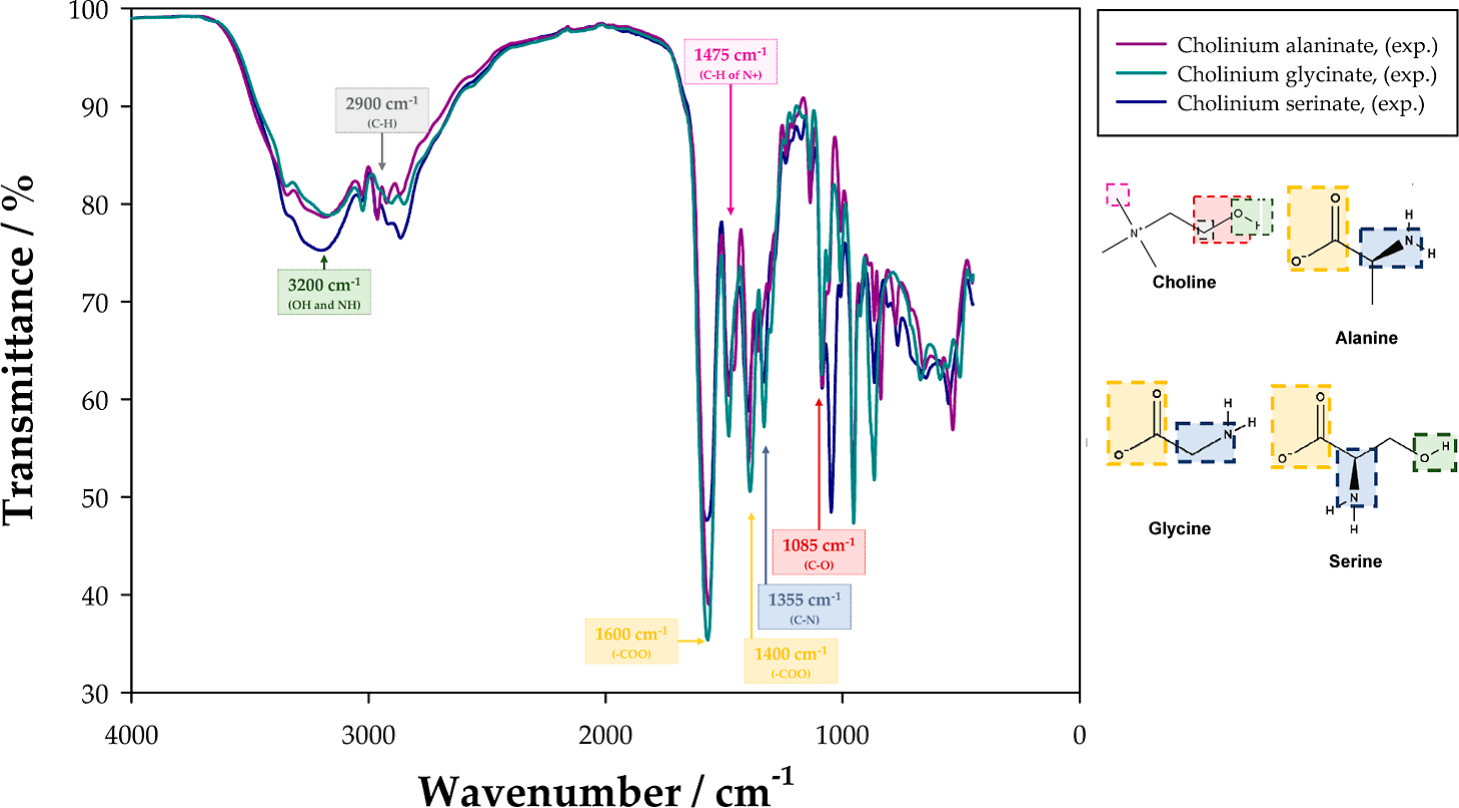
Experimental FTIR spectra of the synthesized [Ch][AA] ILs.

The molecular structures of the synthesized CAAILs, namely, cholinium alaninate ([Ch][Ala]), cholinium glycinate ([Ch][Gly]), and cholinium serinate ([Ch][Ser]), were verified by FTIR spectroscopy, as Figure 2 shows. Overall, the obtained peaks are very similar, such as the one at 3200 cm^–1^, which is caused by the O–H stretching of choline and by the N–H stretching of the amino acids.^31^ However, this peak presents a smaller transmittance in [Ch][Ser] due to an additional O–H bond, while the other CAAILs exhibit similar values between each other. Moreover, other common strong bands are observed at approximately 1600 and 1400 cm^–1^, which correspond, respectively, to the asymmetric and symmetric stretches of the carboxylate group (RCOO^–^).^32^ On the other hand, at ∼1355 and ∼1085 cm^–1^, the common C–N stretching vibration of the amino acid and the C–O stretching of the primary alcohol of choline are notorious,^32^ and, at ∼1475 cm^–1^, the presence of methyl groups of ammonium is evidenced.

After having checked the chemical structures of the synthesized [Ch][AA] ILs, an extra validation was carried out by comparing the experimental FTIR spectra with IR predictions from computational chemistry, as Figures S1–S3, in the Supporting Information, show. Even though peak relative intensities from FTIR are not comparable to the ones from IR obtained using computational chemistry, a general agreement was observed in the peak location, further validating the chemical structure of the synthesized ILs.

**Figure 3:**
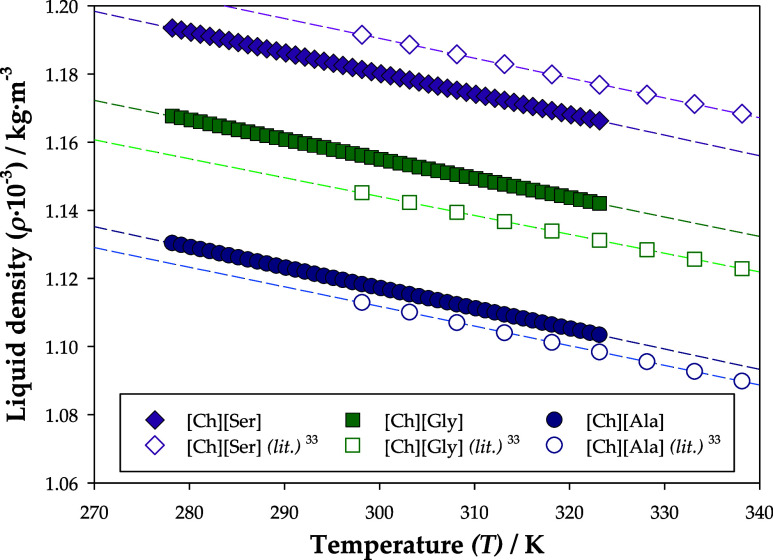
Liquid density from this work and from literature^33^ for [Ch][Ala], [Ch][Gly], and [Ch][Ser], as a function of temperature, at 0.1 MPa. The data regressions from this work follow the equations: *ρ*_[Ch][Ala]_ (kg·m^–3^) = 1297.0–0.5991·*T*(K), *ρ*_[Ch][Gly]_ (kg·m^–3^) = 1326.2–0.5701·*T*(K), and *ρ*_[Ch][Ser]_ (kg·m^–3^) = 1361.8–0.6052·*T*(K), with determination coefficients of 0.99997, 0.99995, and 0.99998, respectively.

Concerning the liquid density (*ρ*) measurements, these were carried out from 278.15 to 323.15 K at 0.1 MPa. The obtained data are reported in Table S1, in the Supporting Information, and compared with the available literature^33^ in Figure 3. In these comparisons, a maximum error of 1% was observed, validating the chemical syntheses of the CAAILs. [Ch][Gly] and [Ch][Ala] presented slightly higher liquid densities than the ones reported in literature, while for [Ch][Ser] the opposite was verified, as previously observed in a previous work.^20^ These variations are probably due to differences in purity or water contamination during the experimental assays.

Moreover, as Figure 3 shows, the liquid densities of the CAAILs follow the order [Ch][Ser] > [Ch][Gly] > [Ch][Ala]. The presence of a hydroxyl group (−OH) in Serine, as seen in Figure 2, increases association (hydrogen bonding), which contributes to a more dense state of [Ch][Ser] molecules. On the other hand, the extra methyl group (−CH_3_) of alanine compared to that of glycine causes steric hindrance and a harder packing of the molecules, which yields larger lattices and a smaller density of [Ch][Ala] compared to [Ch][Gly].

### 3.2 Influence of pH on UV–vis Absorbance

**Figure 4:**
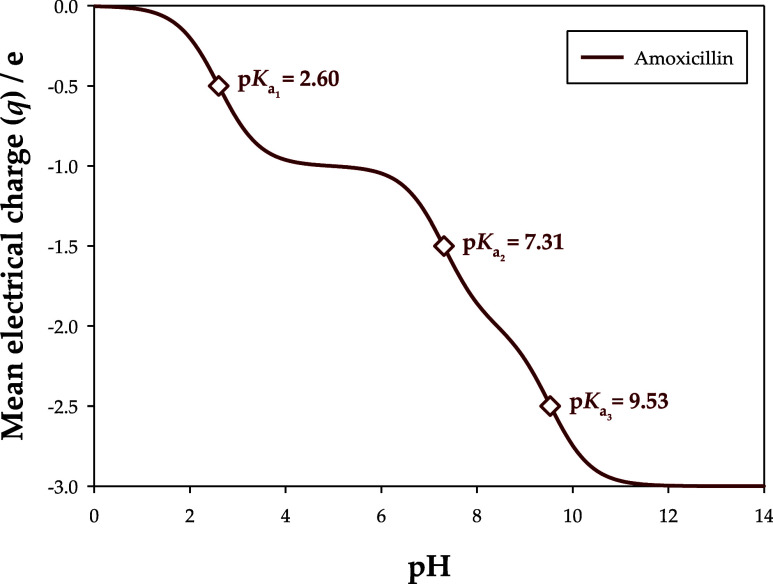
Calculated mean electrical charge (*q*) of amoxicillin as a function of pH. *e* is the electrical charge of an electron (1.6021 × 10^–9^ C).

The molecular structure and chemical conformation of amoxicillin are affected by the donation of protons, which are translated by its acid dissociation constants (p*K*_a_): 2.60, 7.31, and 9.53.^34^ These changes at the molecular level can alter the UV–vis absorbance spectra, making the experimental quantification of labile species very challenging. Depending on the number of p*K*_a_ values a chemical component has, it may show chemical structures with different electric charges, which are commonly referred to as chemical stages.^30^ The weighted mean of the chemical stages with their respective fractions at each pH yields the mean electrical charge (*q*) of the chemical component, as Figure 4 shows for amoxicillin. The full data are presented in Table S2, in the Supporting Information, and it is notorious that, depending on pH, amoxicillin can present four different chemical stages, with mean electrical charges (*q*) of 0, −1, −2, or −3*e*.

The UV–vis absorbance spectra of amoxicillin were studied at different pH values to promote a more accurate quantification of this pharmaceutical. To ease comparison, these spectra were normalized using eq 7, which considers the amount of added pH adjusters (droplets of 0.5 M aqueous solutions of NaOH or CH_3_COOH) and is valid for very dilute solutions. In this equation, the absorbance spectrum at pH = 12.22 was taken as reference given that it is closer to the characteristic pH values of the studied ATPSs, as seen in Table 2.
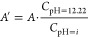
7where *A* is the experimental UV–vis absorbance for a given wavelength (λ), *A*′ is the normalized absorbance, *C*_pH=12.22_ is the reference concentration at pH = 12.22, and *C*_pH=*i*_ is the concentration of amoxicillin in the solution with pH = *i*.

**Figure 5:**
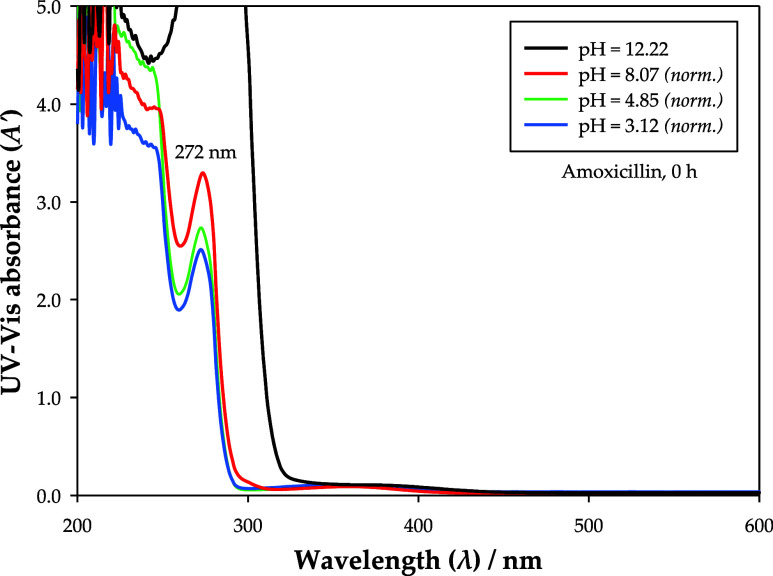
Effect of pH on the UV–vis absorbance spectra of amoxicillin (∼1.70 × 10^–3^ g·mL^–1^) at 298.15 K and 0.1 MPa. Some spectra were normalized using eq 7.

As Figure 5 illustrates, the chemical stages verified at the pH values of 3.12 (*q* ≈ −0.77 *e*), 4.85 (*q* ≈ −1.00 *e*), 8.07 (*q* ≈ −1.88 *e*), and 12.22 (*q* ≈ −3.00 *e*) exhibited different UV–vis absorbance spectra, with an increase in absorbance being observed with growing pH values. This implies that different calibration curves are required for the proper quantification of amoxicillin in systems with very distinct pH values. Nevertheless, as seen in Table 2, high pH values (>12) are expected for all ATPSs in this work, for which only one calibration curve was needed (λ = 272 nm, pH = 12). Furthermore, the UV–vis spectrum was found to be stable with time at this pH since no changes were noticed after 3 days.

### 3.3 Extraction of Amoxicillin

The partition of amoxicillin was conducted in three different ATPSs based on the CAAILs cholinium alaninate ([Ch][Ala]), cholinium glycinate ([Ch][Gly]), or cholinium serinate ([Ch][Ser]) and on the inorganic salt dipotassium hydrogen phosphate (K_2_HPO_4_). The liquid–liquid equilibria of these ATPSs were determined in the previous work of the research group,^29^ as seen in Table 2. After the top and bottom phases were separated, mass, pH, and liquid density were measured for each phase, as Table 3 shows.

**Table 3:** Experimental Mass (*m*), Phase Mass Loss (*L*_m_), Liquid Density (ρ), and pH of Each Phase for the Extraction of Amoxicillin Using the ATPSs {[Ch][Ala] or [Ch][Gly] or [Ch][Ser] (1) + K_2_HPO_4_ (2) + Water (3)} at *T* = 298.15 K and *P* = 0.1 MPa^a^

tie-line	phase	*m* / g	*L*_m_ / %	ρ / kg·m^–^^3^	pH
{[Ch][Ala] (1) + K_2_HPO_4_ (2) + Water (3)}					
1	top	8.9386	–0.26	1113.70	12.12
	bottom	10.9114		1692.26	11.88
2	top	10.3585	–0.43	1113.53	12.09
	bottom	9.2415		1654.36	11.82
3	top	12.8200	–0.34	1115.07	12.02
	bottom	6.7800		1594.63	11.86
4	top	17.0177	–0.98	1129.46	12.03
	bottom	3.1123		1472.75	11.97
{[Ch][Gly] (1) + K_2_HPO_4_ (2) + Water (3)}					
1	top	8.3104	–0.20	1143.23	12.25
	bottom	11.4612		1786.15	12.35
2	top	9.2918	–0.44	1138.61	11.76
	bottom	9.7126		1706.52	12.38
3	top	11.4542	–0.37	1138.65	12.32
	bottom	7.3598		1630.63	12.05
4	top	15.5136	–0.98	1153.64	12.21
	bottom	3.3168		1539.01	11.45
{[Ch][Ser] (1) + K_2_HPO_4_ (2) + Water (3)}					
1	top	7.9342	–0.73	1143.68	12.88
	bottom	11.7016		1751.78	13.38
2	top	8.4186	–0.23	1143.75	12.38
	bottom	10.2868		1659.64	12.33
3	top	11.1520	–0.83	1154.09	12.33
	bottom	7.5478		1576.23	12.34
4	top	12.1628	–0.87	1157.31	12.32
	bottom	6.5028		1541.37	12.09

a The standard measurement uncertainties (*u*) are *u*(*m*) = 10^–4^ g, *u*(*ρ*) = 0.03 kg·m^–3^, *u*(pH) = 10^–2^, *u*(*T*) = 0.01 K, and *u*(*P*) = 2 kPa.

As expected, liquid densities were higher on the bottom phases than on the top phases. In addition, the obtained pH values were alike in top and bottom phases, remaining very similar in the different tie-lines, which ensures similar mean electrical charges (*q*) of amoxicillin and enables the usage of a single calibration curve (determined at pH ≅ 12 and λ = 272 nm).

To evaluate solute migration between the bottom (salt-rich) and top (CAAIL-rich) phases, the partition or distribution coefficients (*K*) were calculated in each feed composition using eq 2. Moreover, given that the main goal of this work was to extract amoxicillin from water, the extraction efficiency (*E*), which refers to the percentage of solute recovered in the top phase, was also calculated using eq 6, as Table 4 shows. These performance indicators were validated by calculating the solute losses in quantification (*L*_s_), which were found to be lower than 5% in mass.

**Table 4:** Tie-Line Length, Amoxicillin Mass Fraction (*w*_Amox_), Partition Coefficient (*K*), Solute Loss in Quantification (*L*_s_), and Extraction Efficiency (*E*) for Each Tie-Line Composition in the Extraction of Amoxicillin Using the ATPSs {[Ch][Ala] or [Ch][Gly] or [Ch][Ser] (1) + K_2_HPO_4_ (2) + Water (3)} at *T* = 298.15 K and *P* = 0.1 MPa^a^

tie-line	TLL / %	*w*_Amox_	*K*	*L*_s_ / %	*E* / %
{[Ch][Ala] (1) + K_2_HPO_4_ (2) + water (3)}					
1	82.179	2.24 × 10^–^^4^	(5 ± 1)·101	–2.47	96 ± 1
		2.97 × 10^–^^6^			
2	78.036	1.87 × 10^–^^4^	31 ± 6	–3.13	95 ± 1
		4.09 × 10^–^^6^			
3	69.754	1.50 × 10^–^^4^	12 ± 2	–4.39	93 ± 1
		8.94 × 10^–^^6^			
4	57.642	1.12 × 10^–^^4^	4.4 ± 0.2	–1.65	95 ± 1
		1.95 × 10^–^^5^			
{[Ch][Gly] (1) + K_2_HPO_4_ (2) + water (3)}					
1	95.127	2.33 × 10^–^^4^	(16 ± 6)·101	–2.39	97 ± 2
		9.44 × 10^–^^7^			
2	85.053	2.10 × 10^–^^4^	(5 ± 2)·101	–1.78	97 ± 1
		2.58 × 10^–^^6^			
3	73.637	1.66 × 10^–^^4^	16 ± 2	–2.74	95 ± 1
		7.45 × 10^–^^6^			
4	58.908	1.23 × 10^–^^4^	4.3 ± 0.2	–2.05	94 ± 1
		2.14 × 10^–^^5^			
{[Ch][Ser] (1) + K_2_HPO_4_ (2) + water (3)}					
1	102.347	4.38 × 10^–^^5^	4.47 ± 0.5	–2.55	80 ± 1
		6.40 × 10^–^^6^			
2	82.944	1.76 × 10^–^^4^	2.65 ± 0.06	–2.43	74 ± 1
		4.57 × 10^–^^5^			
3	69.028	1.30 × 10^–^^4^	1.49 ± 0.03	–3.57	72 ± 1
		6.36 × 10^–^^5^			
4	65.004	1.23 × 10^–^^4^	1.24 ± 0.03	–1.12	75 ± 1
		7.41 × 10^–^^5^			

a The standard measurement uncertainties (*u*) are *u*(*T*) = 0.01 K and *u*(*P*) = 2 kPa.

**Figure 6:**
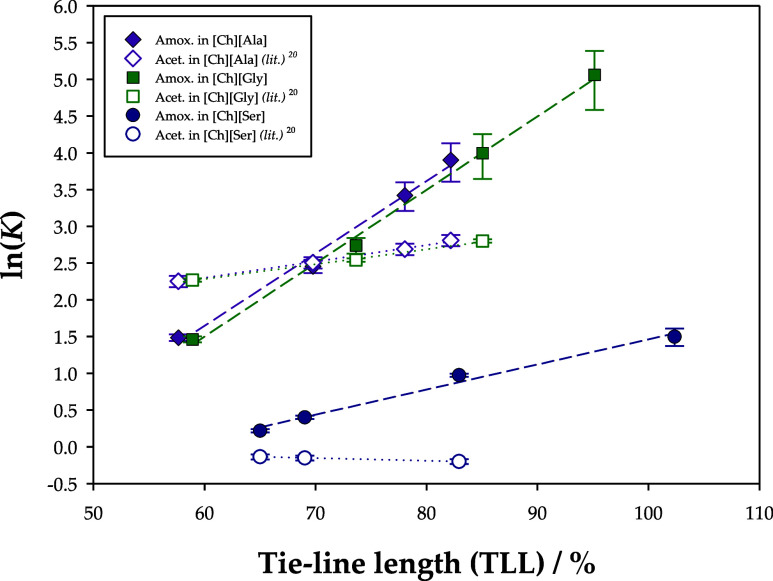
Relation of the TLL with the natural logarithm of the partition coefficient (ln(*K*)) for the extraction of amoxicillin in the systems {[Ch][Ala] or [Ch][Gly] or [Ch][Ser] (1) + K_2_HPO_4_ (2) + water (3)} at *T* = 298.15 K and *P* = 0.1 MPa. The experimental results were compared with data from literature^20^ involving the extraction of acetaminophen (paracetamol) in the same ATPSs.

If only the common dominium is considered, the ATPS {[Ch][Ala] (1) + K_2_HPO_4_ (2) + water (3)} presented the highest partition coefficients (*K*), while {[Ch][Ser] (1) + K_2_HPO_4_ (2) + water (3)} showed the smallest. In fact, even though all studied systems presented partition coefficients above unity, indicating a more favorable migration of amoxicillin to the top phase, for tie-lines number 3 and 4, in the ATPS based on [Ch][Ser], an almost equal solute distribution is observed between the liquid phases (*K* ≅ 1), which obviously undermines the practical application of feed compositions with shorter tie-line length (TLL). To better study this phenomenon, the natural logarithms of this performance indicator were plotted as a function of the TLLs in Figure 6.

In a ternary phase diagram, the TLL refers to the distance between the two points that signal the composition of the top and bottom phases in a liquid–liquid equilibrium. As expected, longer tie-lines provided higher partition coefficients (*K*), so systems with an increased CAAIL concentration in the top phase and increased salt concentration in the bottom phase more significantly favored solute migration to the top phase. This is probably due to a more accentuated difference in properties such as conductivity, hydrophobicity, and density between the phases. For this reason, the observed partition coefficients for the system based on [Ch][Gly] (maximum TLL of 95.127%) outperformed [Ch][Ala] (maximum TLL of 82.179%). Nevertheless, the overall behavior of the partition coefficients with growing TLL is very similar in [Ch][Ala] and [Ch][Gly] due to their resemblance of chemical properties and structure.

Additionally, Figure 6 also compares the obtained partition coefficients with the ones observed for acetaminophen (or paracetamol, *i.e.*, a common analgesic and antipyretic) in a previous work^20^ of the research group which used the same ATPSs and extraction methodology. Similarly to amoxicillin, the partition coefficients for acetaminophen followed the order [Ch][Ala] > [Ch][Gly] > [Ch][Ser], but a more notorious difference was noticed between [Ch][Ser] and the other ATPSs. Once again, the reported results for the extraction of acetaminophen in [Ch][Ala] and [Ch][Gly] were very similar due to the resemblance of the chemical structures of these CAAILs. Moreover, amoxicillin generally achieved more promising values of this performance indicator, for which these ATPSs are more favorable for the removal of this pharmaceutical pollutant.

**Figure 7:**
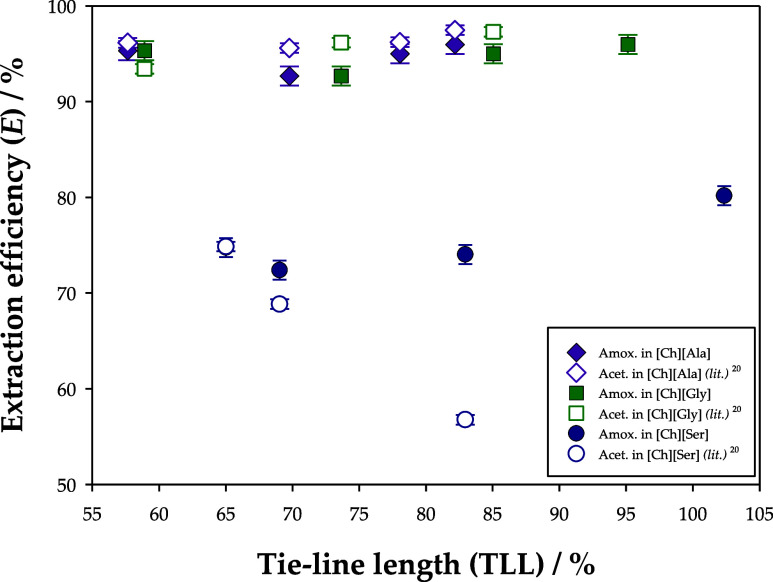
Relation of the TLL with the extraction efficiency (*E*) for the extraction of amoxicillin in the systems {[Ch][Ala] or [Ch][Gly] or [Ch][Ser] (1) + K_2_HPO_4_ (2) + water (3)} at *T* = 298.15 K and *P* = 0.1 MPa. The experimental results were compared with data from literature^20^ involving the extraction of acetaminophen (paracetamol) in the same ATPSs.

Regarding the extraction efficiencies (*E*), both amoxicillin and acetaminophen followed the order [Ch][Ala] ≅ [Ch][Gly] > [Ch][Ser], as Figure 7 shows. Furthermore, TLL did not present a significant effect on this performance indicator, and very similar values were attained for amoxicillin and acetaminophen in all ATPSs except for the one based on [Ch][Ser]. Even though these APIs possess negative mean electrical charges (*q* = −3.00 *e* for amoxicillin and *q* = −1.00 *e* for acetaminophen, at pH = 12), less polar ILs provided more favorable extractive media, for which these electrical charges appear to be rather distributed throughout the molecules.

It must be noted that extraction efficiencies are extremely sensitive to the ratio of masses between the phases of the ATPSs, so, in this case, feed compositions that yield larger top phases will cause higher recoveries of the pharmaceutical pollutant. Therefore, their interpretation alone is insufficient for the correct assessment of the extractive capacity of an ATPS and should always be coupled with the determination of the partition coefficients. Yet, the maximization of the extraction efficiencies by adjusting the feed compositions is a paramount step in the downstream optimization of extractive processes.

## 4 Conclusions

In this work, the performance of three ATPSs based on CAAILs ([Ch][Ala], [Ch][Gly], or [Ch][Ser]) and an inorganic salt (K_2_HPO_4_) was assessed in the removal of amoxicillin from water at 298.15 K and 0.1 MPa. All the studied ATPSs provided partition coefficients larger than unity, evidencing an affinity of amoxicillin toward the CAAIL-rich phase. Moreover, the extraction was favored by larger TLL and by less polar ILs, achieving maximum partition coefficients (*K*) and extraction efficiencies (*E*) of *K* = (16 ± 6)·10^1^ and *E* / % = 97 ± 2, respectively, for {[Ch][Gly] (1) + K_2_HPO_4_ (2) + water (3)} with TLL = 95.127%.

This way, this work provided preliminary data on the effectiveness of green ILs in the extraction of amoxicillin, which may become vital for the future development of more sustainable approaches to tackle antibiotic contamination in water bodies. Moreover, the obtained performance indicators were checked by calculating the solute losses in quantification (<5% in mass) and computational chemistry was shown to be a powerful tool for the validation of molecular structures during chemical synthesis.

## Supporting Information Available

Predicted IR spectra using computational chemistry for the studied CAAILs and calculated mean electrical charges (*q*) and relative abundances (*x*) of the different stages of amoxicillin (PDF)

## References

[ref1] Jung Y. J.; Kim W. G.; Yoon Y.; Kang J. W.; Hong Y. M.; Kim H. W. Removal of amoxicillin by UV and UV/H2O2 processes. Sci. Total Environ. 2012, 420, 160–167. 10.1016/j.scitotenv.2011.12.011. 22326316

[ref2] Kummerer K. Antibiotics in the aquatic environment-a review-part I. Chemosphere 2009, 75, 417–434. 10.1016/j.chemosphere.2008.11.086. 19185900

[ref3] Li D.; Yang M.; Hu J.; Zhang Y.; Chang H.; Jin F. Determination of penicillin G and its degradation products in a penicillin production wastewater treatment plant and the receiving river. Water Res. 2008, 42, 307–317. 10.1016/j.watres.2007.07.016. 17675133

[ref4] Sodhi K. K.; Kumar M.; Singh D. K. Insight into the amoxicillin resistance, ecotoxicity, and remediation strategies. J. Water Process Eng. 2021, 39, 101858 10.1016/j.jwpe.2020.101858.

[ref5] Lima L. M.; Silva B.; Barbosa G.; Barreiro E. J. β-lactam antibiotics: An overview from a medicinal chemistry perspective. Eur. J. Med. Chem. 2020, 208, 112829 10.1016/j.ejmech.2020.112829. 33002736

[ref6] Aryee A. A.; Han R.; Qu L. Occurrence, detection and removal of amoxicillin in wastewater: A review. J. Cleaner Prod. 2022, 368, 133140 10.1016/j.jclepro.2022.133140.

[ref7] Rodriguez-Mozaz S.; Vaz-Moreira I.; Giustina S. V. D.; Llorca M.; Barcelo D.; Schubert S.; Berendonk T. U.; Michael-Kordatou I.; Fatta-Kassinos D.; Martinez J. L.; Elpers C.; Henriques I.; Jaeger T.; Schwartz T.; Paulshus E.; O’Sullivan K.; Parnanen K. M. M.; Virta M.; Do T. T.; Walsh F.; Manaia C. M. Antibiotic residues in final effluents of European wastewater treatment plants and their impact on the aquatic environment. Environ. Int. 2020, 140, 105733 10.1016/j.envint.2020.105733. 32353669

[ref8] Kovalakova P.; Cizmas L.; McDonald T. J.; Marsalek B.; Feng M.; Sharma V. K. Occurrence and toxicity of antibiotics in the aquatic environment: A review. Chemosphere 2020, 251, 126351 10.1016/j.chemosphere.2020.126351. 32443222

[ref9] Gotvajn A. Ž.; Rozman U.; Antončič T.; Urbanc T.; Vrabeĺ M.; Derco J. Fe2+ and UV Catalytically Enhanced Ozonation of Selected Environmentally Persistent Antibiotics. Processes 2021, 9, 521 10.3390/pr9030521.

[ref10] Ay F.; Kargi F. Advanced oxidation of amoxicillin by Fenton’s reagent treatment. J. Hazard. Mater. 2010, 179, 622–627. 10.1016/j.jhazmat.2010.03.048. 20363555

[ref11] Alalm M. G.; Tawfik A.; Ookawara S. Degradation of four pharmaceuticals by solar photo-Fenton process: Kinetics and costs estimation. J. Environ. Chem. Eng. 2015, 3, 46–51. 10.1016/j.jece.2014.12.009.

[ref12] Farrugia C.; Di Mauro A.; Lia F.; Zammit E.; Rizzo A.; Privitera V.; Impellizzeri G.; Buccheri M. A.; Rappazzo G.; Grech M.; Refalo P.; Abela S. Suitability of Different Titanium Dioxide Nanotube Morphologies for Photocatalytic Water Treatment. Nanomaterials 2021, 11, 708 10.3390/nano11030708. 33799849 PMC7998466

[ref13] Ezugbe E. O.; Rathilal S. Membrane Technologies in Wastewater Treatment: A Review. Membranes 2020, 10, 89 10.3390/membranes10050089. 32365810 PMC7281250

[ref14] Iqbal M.; Tao Y.; Xie S.; Zhu Y.; Chen D.; Wang X.; Huang L.; Peng D.; Sattar A.; Shabbir M. A.; Hussain H. I.; Ahmed S.; Yuan Z. Aqueous two-phase system (ATPS): an overview and advances in its applications. Biol. Proced. Online 2016, 18, 18 10.1186/s12575-016-0048-8. 27807400 PMC5084470

[ref15] Zhang Q.; Sang Z.; Li Q.; Gong J.; Shi R.; Zhang B.; Zhang Z.; Li S.; Yang X. Palladium(II) extraction from acidic chloride media using an ionic liquid-based aqueous two-phase system (IL-ATPS) in the presence of dipotassium hydrogen phosphate salting-out agent and reductive stripping with hydrazine hydrate to recover palladium metal. Hydrometallurgy 2023, 216, 106017 10.1016/j.hydromet.2022.106017.

[ref16] Scurto A. M.; Newton E.; Weikel R. R.; Draucker L.; Hallett J.; Liotta C. L.; Leitner W.; Eckert C. A. Melting Point Depression of Ionic Liquids with CO2: Phase Equilibria. J. Chem. Eng. Data 2008, 47, 493–501. 10.1021/ie070312b.

[ref17] Song J. Research Progress of Ionic Liquids as Lubricants. ACS Omega 2021, 6, 29345–29349. 10.1021/acsomega.1c04512. 34778607 PMC8581986

[ref18] Latini G.; Signorile M.; Crocellà V.; Bocchini S.; Pirri C. F.; Bordiga S. Unraveling the CO2 reaction mechanism in bio-based amino-acid ionic liquids by operando ATR-IR spectroscopy. Catal. Today 2019, 336, 148–160. 10.1016/j.cattod.2018.12.050.

[ref19] Petkovic M.; Ferguson J. L.; Gunaratne H. Q. N.; Ferreira R.; Leitão M. C.; Seddon K. R.; Rebelo L. P. N.; Pereira C. S. Novel biocompatible cholinium-based ionic liquids—toxicity and biodegradability. Green Chem. 2010, 12, 643–649. 10.1039/b922247b.

[ref20] Barroca L. R.; Velho P.; Macedo E. A. Removal of Acetaminophen (Paracetamol) from Water Using Aqueous Two-Phase Systems (ATPSs) Composed of Choline-Amino Acid Ionic Liquids. J. Chem. Eng. Data 2024, 69, 215–226. 10.1021/acs.jced.3c00602.

[ref21] Li Y.; Yang F.; Li Y.; Cai M.; Li H.; Fan X.; Zhu M. Choline amino acid ionic Liquids: A novel green potential lubricant. J. Mol. Liq. 2022, 360, 119539 10.1016/j.molliq.2022.119539.

[ref22] Assis R. C.; Mageste A. B.; de Lemos L. R.; Orlando R. M.; Rodrigues G. D. Application of aqueous two-phase systems for the extraction of pharmaceutical compounds from water samples. J. Mol. Liq. 2020, 301, 112411 10.1016/j.molliq.2019.112411.

[ref23] Al-Saidi S.; Mjalli F. S.; Al-Azzawi M.; Abutarboosh B.; AlSaadi M. A.; Al-Wahaibi T. Amoxicillin removal from medical wastewater using an eco-friendly aqueous two-phase extraction system. Sep. Sci. Technol. 2023, 58, 61–74. 10.1080/01496395.2022.2102998.

[ref24] Pinho S. P.; Macedo E. A. Solubility of NaCl, NaBr, and KCl in Water, Methanol, Ethanol, and Their Mixed Solvents. J. Chem. Eng. Data 2005, 50, 29–32. 10.1021/je049922y.

[ref25] Velho P.; Sousa E.; Macedo E. A. Extraction of Salicylic Acid Using Sustainable ATPSs and Respective Immobilization as API-IL at Small Pilot Scale. J. Chem. Eng. Data 2024, 10.1021/acs.jced.3c00594.

[ref26] Frisch M. J.; Trucks G. W.; Schlegel H. B.; Scuseria G. E.; Robb M. A.; Cheeseman J. R.; Scalmani G.; Barone V.; Mennucci B.; Petersson G. A.; Nakatsuji H.; Caricato M.; Li X.; Hratchian H. P.; Izmaylov A. F.; Bloino J.; Zheng G.; Sonnenberg J. L.; Hada M.; Ehara M.; Toyota K.; Fukuda R.; Hasegawa J.; Ishida M.; Nakajima T.; Honda Y.; Kitao O.; Nakai H.; Vreven T.; Jr J. A. M.; Peralta J. E.; Ogliaro F.; Bearpark M.; Heyd J. J.; Brothers E.; Kudin K. N.; Staroverov V. N.; Kobayashi R.; Normand J.; Raghavachari K.; Rendell A.; Burant J. C.; Iyengar S. S.; Tomasi J.; Cossi M.; Rega N.; Millam J. M.; Klene M.; Knox J. E.; Cross J. B.; Bakken V.; Adamo C.; Jaramillo J.; Gomperts R.; Stratmann R. E.; Yazyev O.; Austin A. J.; Cammi R.; Pomelli C.; Ochterski J. W.; Martin R. L.; Morokuma K.; Zakrzewski V. G.; Voth G. A.; Salvador P.; Dannenberg J. J.; Dapprich S.; Daniels A. D.; Farkas O. ¨.; Foresman J. B.; Ortiz J. V.; Cioslowski J.; Gaussian D. J. F. Gaussian 09, Revision D.01; Gaussian, Inc.: Wallingford, CT, 2009.

[ref27] Becke A. D. Density-functional thermochemistry. III. The role of exact exchange. J. Chem. Phys. 1993, 98, 5648–5652. 10.1063/1.464913.

[ref28] Francl M. M.; Pietro W. J.; Hehre W. J.; Binkley J. S.; Gordon M. S.; DeFrees D. J.; Pople J. A. Self-consistent molecular orbital methods. XXIII. A polarization-type basis set for second-row elements. J. Chem. Phys. 1982, 77, 3654–3665. 10.1063/1.444267.

[ref29] Gómez E.; Requejo P. F.; Tojo E.; Macedo E. A. Recovery of flavonoids using novel biodegradable choline amino acids ionic liquids based ATPS. Fluid Phase Equilib. 2019, 493, 1–9. 10.1016/j.fluid.2019.03.024.

[ref30] Rebelo C. S.; Velho P.; Macedo E. A. Partition Studies of Resveratrol in Low-Impact ATPS for Food Supplementation. Ind. Eng. Chem. Res. 2024, 63, 2885–2894. 10.1021/acs.iecr.3c03969.

[ref31] Li Q.; Liu W.; Zhu X. Green choline amino acid ionic liquids aqueous two-phase extraction coupled with synchronous fluorescence spectroscopy for analysis naphthalene and pyrene in water samples. Talanta 2020, 219, 121305 10.1016/j.talanta.2020.121305. 32887046

[ref32] Foulet A.; Ghanem O. B.; El-Harbawi M.; Lévêque J. M.; Mutalib M. A.; Yin C.-Y. Understanding the physical properties, toxicities and anti-microbial activities of choline-amino acid-based salts: Low-toxic variants of ionic liquids. J. Mol. Liq. 2016, 221, 133–138. 10.1016/j.molliq.2016.05.046.

[ref33] Tao D.-J.; Cheng Z.; Chen F.-F.; Li Z.-M.; Hu N.; Chen X.-S. Synthesis and Thermophysical Properties of Biocompatible Cholinium-Based Amino Acid Ionic Liquids. J. Chem. Eng. Data 2013, 58, 1542–1548. 10.1021/je301103d.

[ref34] Bergström C. A. S.; Strafford M.; Lazorova L.; Avdeef A.; Luthman K.; Artursson P. Absorption classification of oral drugs based on molecular surface properties. J. Med. Chem. 2003, 46, 558–570. 10.1021/jm020986i. 12570377

