# Supplementary material for: Water Purification Using Choline-Amino Acid Ionic Liquids: Removal of Amoxicillin
Source: Ind Eng Chem Res. 2024 May 21;63(23):10427–35. doi: 10.1021/acs.iecr.4c01002 (PMC11178035; doi:10.1021/acs.iecr.4c01002)
Supplement: Supplementary file 1 — ie4c01002_si_001.pdf [file ie4c01002_si_001.pdf]

Supplementary Materials:

Water purification using choline-amino acid ionic liquids: removal of amoxicillin

Pedro Velho [1, 2, a, *], Catarina Lopes [1, 2, b], Eugénia A. Macedo [1, 2, c, *]

[1] LSRE-LCM – Laboratory of Separation and Reaction Engineering - Laboratory of Catalysis and Materials, Faculty of Engineering, University of Porto, Rua Dr. Roberto Frias, 4200-465 Porto, Portugal.

[2] ALiCE – Associate Laboratory in Chemical Engineering, Faculty of Engineering, University of Porto, Rua Dr. Roberto Frias, 4200-465 Porto, Portugal.

[a] e-mail: velho@fe.up.pt

[b] e-mail: catarinalopes@fe.up.pt

[c] e-mail: eamacedo@fe.up.pt

[*] Corresponding authors.

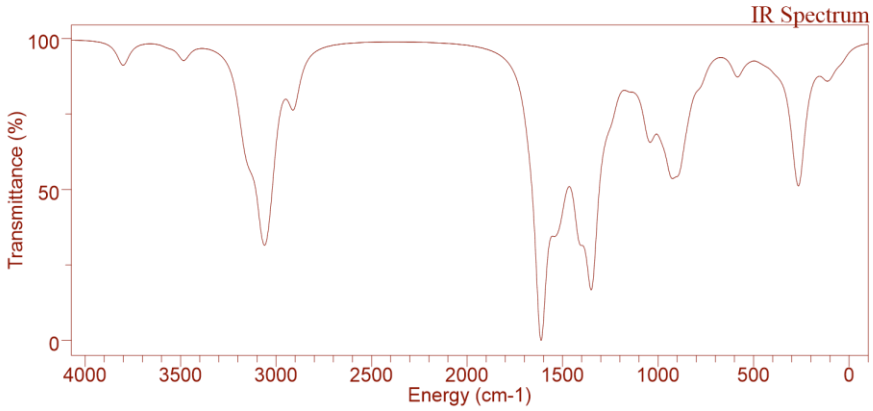

Fig. S1 – Predicted infrared (IR) spectrum for cholinium alaninate ([Ch][Ala]) using Density Functional Theory (DFT) with the B3LYP hybrid functional [1] and the 6-311+G(*,*) basis set [2].

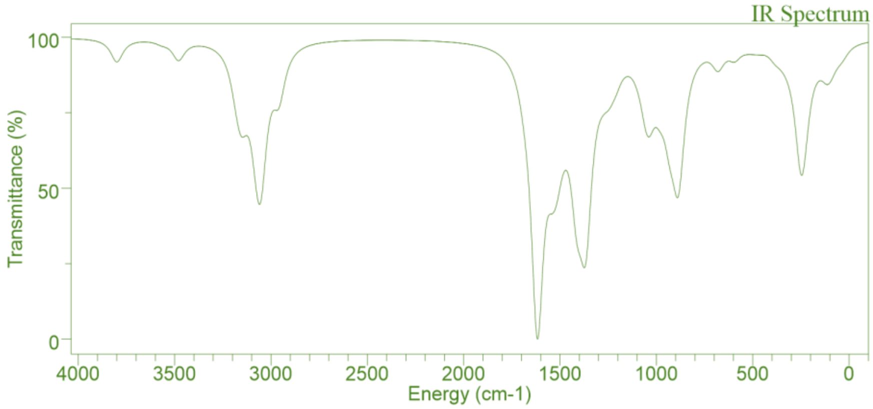

Fig. S2 – Predicted infrared (IR) spectrum for cholinium glycinate ([Ch][Gly]) using Density Functional Theory (DFT) with the B3LYP hybrid functional [1] and the 6-311+G(*,*) basis set [2].

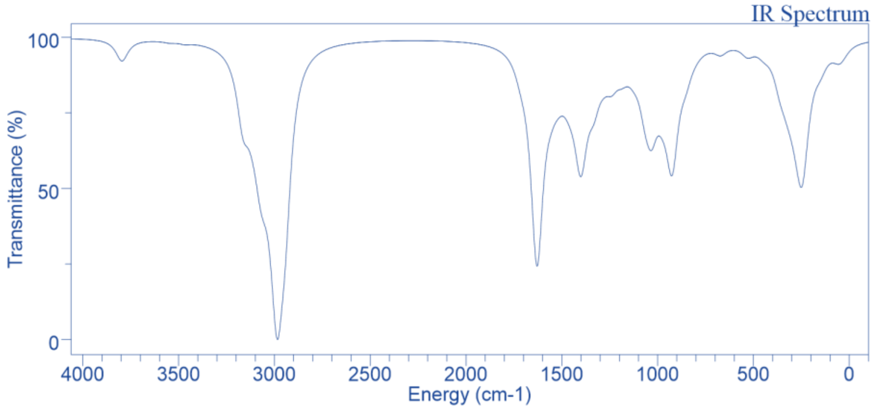

Fig. S3 – Predicted infrared (IR) spectrum for cholinium serinate ([Ch][Ser]) using Density Functional Theory (DFT) with the B3LYP hybrid functional [1] and the 6-311+G(*,*) basis set [2].

Table S1 – Liquid density (ρ) with temperature (T) for cholinium alaninate, [Ch][Ala], cholinium glycinate, [Ch][Gly], and cholinium serinate, [Ch][Ser], and respective comparison with literature [3], at P = 0.1 MPa.

	[Ch][Ala]		[Ch][Gly]		[Ch][Ser]	
T / K	*(exp.)*	*(lit.)* [3]	*(exp.)*	*(lit.)* [3]	*(exp.)*	*(lit.)* [3]
278.15	1130.41		1167.74		1193.53	
279.13	1129.83		1167.18		1192.94	
280.13	1129.22		1166.60		1192.33	
281.13	1128.62		1166.02		1191.72	
282.13	1128.01		1165.44		1191.10	
283.13	1127.41		1164.85		1190.49	
284.13	1126.80		1164.27		1189.88	
285.13	1126.19		1163.69		1189.27	
286.13	1125.59		1163.11		1188.65	
287.13	1124.98		1162.53		1188.04	
288.12	1124.38		1161.95		1187.43	
289.13	1123.77		1161.37		1186.82	
290.13	1123.17		1160.80		1186.21	
291.13	1122.56		1160.22		1185.60	
292.13	1121.96		1159.64		1184.99	
293.13	1121.36		1159.07		1184.38	
294.13	1120.71		1158.49		1183.78	
295.13	1120.11		1157.91		1183.17	
296.13	1119.51		1157.33		1182.56	
297.13	1118.91		1156.75		1181.96	
298.13	1118.32	1112.94	1156.18	1145.20	1181.35	1191.51
299.13	1117.72		1155.60		1180.74	
300.13	1117.13		1155.03		1180.11	
301.13	1116.53		1154.47		1179.51	
302.13	1115.94		1153.90		1178.90	
303.13	1115.35	1110.07	1153.34	1142.31	1178.30	1188.65
304.13	1114.75		1152.77		1177.69	
305.13	1114.16		1152.21		1177.09	
306.13	1113.56		1151.64		1176.49	
307.13	1112.97		1151.08		1175.89	
308.13	1112.38	1107.01	1150.52	1139.43	1175.30	1185.79
309.13	1111.78		1149.96		1174.70	
310.13	1111.19		1149.39		1174.10	
311.13	1110.59		1148.83		1173.50	
312.13	1110.00		1148.27		1172.90	
313.13	1109.40	1104.04	1147.71	1136.70	1172.31	1182.90
314.13	1108.81		1147.15		1171.71	
315.13	1108.21		1146.58		1171.11	
316.13	1107.62		1146.02		1170.51	
317.13	1107.02		1145.46		1169.91	
318.13	1106.43	1101.21	1144.89	1133.97	1169.31	1179.92
319.13	1105.83		1144.33		1168.71	
320.14	1105.24		1143.77		1168.11	
321.14	1104.64		1143.21		1167.51	
322.14	1104.05		1142.64		1166.91	
323.14	1103.45	1098.37	1142.08	1131.22	1166.31	1176.87
328.15		1095.54		1128.46		1173.97
333.15		1092.69		1125.70		1171.20
338.15		1089.85		1122.93		1168.38

[a] The standard measurement uncertainties (u) are: $u(T)$ = 0.01 K, $u(P)$ = 2 kPa and $u(\rho)$ = 0.03 kg·m^{-3}.

Table S2 - Calculated fraction of each chemical stage and mean electrical charge (q) at different pH values for amoxicillin (Amox).

**pH**	$x_{\mathbf{Amox.}^{0}}$	$x_{\mathbf{Amox.}^{-1}}$	$x_{\mathbf{Amox.}^{-2}}$	$x_{\mathbf{Amox.}^{-3}}$	q / **e**
0.00	1.00	0.00	0.00	0.00	0.00
0.25	1.00	0.00	0.00	0.00	0.00
0.50	0.99	0.01	0.00	0.00	-0.01
0.75	0.99	0.01	0.00	0.00	-0.01
1.00	0.98	0.02	0.00	0.00	-0.02
1.25	0.96	0.04	0.00	0.00	-0.04
1.50	0.93	0.07	0.00	0.00	-0.07
1.75	0.88	0.12	0.00	0.00	-0.12
2.00	0.80	0.20	0.00	0.00	-0.20
2.25	0.69	0.31	0.00	0.00	-0.31
2.50	0.56	0.44	0.00	0.00	-0.44
2.75	0.41	0.59	0.00	0.00	-0.59
3.00	0.28	0.72	0.00	0.00	-0.72
3.25	0.18	0.82	0.00	0.00	-0.82
3.50	0.11	0.89	0.00	0.00	-0.89
3.75	0.07	0.93	0.00	0.00	-0.93
4.00	0.04	0.96	0.00	0.00	-0.96
4.25	0.02	0.98	0.00	0.00	-0.98
4.50	0.01	0.99	0.00	0.00	-0.99
4.75	0.01	0.99	0.00	0.00	-1.00
5.00	0.00	0.99	0.00	0.00	-1.00
5.25	0.00	0.99	0.01	0.00	-1.01
5.50	0.00	0.98	0.02	0.00	-1.01
5.75	0.00	0.97	0.03	0.00	-1.03
6.00	0.00	0.95	0.05	0.00	-1.05
6.25	0.00	0.92	0.08	0.00	-1.08
6.50	0.00	0.87	0.13	0.00	-1.13
6.75	0.00	0.78	0.22	0.00	-1.22
7.00	0.00	0.67	0.33	0.00	-1.33
7.25	0.00	0.53	0.46	0.00	-1.47
7.50	0.00	0.39	0.60	0.01	-1.62
7.75	0.00	0.26	0.72	0.01	-1.75
8.00	0.00	0.17	0.81	0.02	-1.86
8.25	0.00	0.10	0.86	0.04	-1.95
8.50	0.00	0.06	0.86	0.08	-2.02
8.75	0.00	0.03	0.83	0.14	-2.11
9.00	0.00	0.02	0.76	0.22	-2.21
9.25	0.00	0.01	0.65	0.34	-2.33
9.50	0.00	0.00	0.52	0.48	-2.48
9.75	0.00	0.00	0.38	0.62	-2.62
10.00	0.00	0.00	0.25	0.75	-2.75
10.25	0.00	0.00	0.16	0.84	-2.84
10.50	0.00	0.00	0.10	0.90	-2.90
10.75	0.00	0.00	0.06	0.94	-2.94
11.00	0.00	0.00	0.03	0.97	-2.97
11.25	0.00	0.00	0.02	0.98	-2.98
11.50	0.00	0.00	0.01	0.99	-2.99
11.75	0.00	0.00	0.01	0.99	-2.99
12.00	0.00	0.00	0.00	1.00	-3.00
12.25	0.00	0.00	0.00	1.00	-3.00
12.50	0.00	0.00	0.00	1.00	-3.00
12.75	0.00	0.00	0.00	1.00	-3.00
13.00	0.00	0.00	0.00	1.00	-3.00
13.25	0.00	0.00	0.00	1.00	-3.00
13.50	0.00	0.00	0.00	1.00	-3.00
13.75	0.00	0.00	0.00	1.00	-3.00
14.00	0.00	0.00	0.00	1.00	-3.00

where $x_{\mathrm{Amox.}^{0}}$, $x_{\mathrm{Amox.}^{-1}}$, $x_{\mathrm{Amox.}^{-2}}$ and $x_{\mathrm{Amox.}^{-3}}$ are the stage fractions of electrical charges equal to 0, -1, -2 and -3 e, respectively. e stands for the elementary charge ($1.60 \cdot 10^{-19}$ C).

References

(1) Becke, A.D. Density-functional thermochemistry. III. The role of exact exchange. *J. Chem. Phys.* **1993**, 98, 5648-5652.
(2) Francl, M.M.; Pietro, W.J.; Hehre, W.J.; Binkley, J.S.; Gordon, M.S.; DeFrees, D.J.; Pople, J.A. Self-consistent molecular orbital methods. XXIII. A polarization-type basis set for second-row elements. *J. Chem. Phys.* **1982**, 77, 3654-3665.
(3) Tao, D.-J.; Cheng, Z.; Chen, F.-F.; Li, Z.-M.; Hu, N.; Chen, X.-S. Synthesis and Thermophysical Properties of Biocompatible Cholinium-Based Amino Acid Ionic Liquids. *J. Chem. Eng. Data* **2013**, 58, 1542-1548.
